# Histone Variant MacroH2A1 Plays an Isoform-Specific Role in Suppressing Epithelial-Mesenchymal Transition

**DOI:** 10.1038/s41598-018-19364-4

**Published:** 2018-01-16

**Authors:** Dayle Q. Hodge, Jihong Cui, Matthew J. Gamble, Wenjun Guo

**Affiliations:** 1 Ruth L. and David S. Gottesman Institute for Stem Cell and Regenerative Medicine Research, Albert Einstein College of Medicine, Bronx, NY 10461 USA; 2 Department of Cell Biology, Albert Einstein College of Medicine, Bronx, NY 10461 USA; 3 Department of Molecular Pharmacology, Albert Einstein College of Medicine, Bronx, NY 10461 USA

## Abstract

Epithelial-Mesenchymal Transition (EMT) is a biological program that plays key roles in various developmental and pathological processes. Although much work has been done on signaling pathways and transcription factors regulating EMT, the epigenetic regulation of EMT remains not well understood. Histone variants have been recognized as a key group of epigenetic regulators. Among them, macroH2A1 is involved in stem cell reprogramming and cancer progression. We postulated that macroH2A1 may play a role in EMT, a process involving reprogramming of cellular states. In this study, we demonstrate that expression of macroH2A1 is dramatically reduced during EMT induction in immortalized human mammary epithelial cells (HMLE). Moreover, ectopic expression of the macroH2A1.1 isoform, but not macroH2A1.2, can suppress EMT induction and reduce the stem-like cell population in HMLE. Interestingly, macroH2A1.1 overexpression cannot revert stable mesenchymal cells back to the epithelial state, suggesting a stage-specific role of macroH2A1.1 in EMT. We further pinpointed that the function of macroH2A1.1 in EMT suppression is dependent on its ability to bind the NAD^+^ metabolite PAR, in agreement with the inability to suppress EMT by macroH2A1.2, which lacks the PAR binding domain. Thus, our work discovered a previously unrecognized isoform-specific function of macroH2A1 in regulating EMT induction.

## Introduction

Epithelial-Mesenchymal Transition (EMT) is a biological program playing key roles in a number of biological processes including embryonic development, wound healing and fibrosis, as well as carcinoma metastasis^1,2^. During EMT, epithelial cells lose their characteristics of apical-basal polarity, reduce expression of intercellular adhesion proteins (such as E-Cadherin and Occludin) with neighboring cells and acquire mesenchymal properties such as: fibroblast-like morphology; expression of N-Cadherin, vimentin and fibronectin; and display increased motility and resistance to apoptosis^2,3^. Though EMT changes cell characteristics between two distinct states, the process is not binary. Rather EMT reflects a broad spectrum of partial EMT states in which cells have various degrees of hybrid epithelial and mesenchymal phenotypes^1,4^. EMT is also a reversible process, in which cells regain epithelial features through mesenchymal-epithelial transition (MET). These dynamic cell fate changes are regulated by a network of complex and often interacting signaling pathways. Understanding the role each of these pathways plays in EMT regulation is crucial to full comprehension of these important biological processes.

EMT is particularly important during the metastasis of epithelial cancers. The vast majority of cancer deaths (approximately 90%) are attributable to complications from dissemination of the tumor, and not the primary carcinoma^5^. Metastasis is determined by the ability of cancer cells to grow and spread beyond the primary tumor to distant organs. Both of these phenomena are predicated on the ability of a carcinoma to change its properties based upon the environment where it resides. EMT and its reverse process MET play critical roles during each of these processes^2,6^. Solid tumors are primarily epithelial and dissociating from the bulk of the tumor, traversing into a vessel, surviving in the bloodstream and establishing a colony elsewhere requires shifting from an epithelial to a mesenchymal phenotype, and then back again. EMT provides the cues necessary to survive these very different environments.

EMT also provides a pathway for the production of cancer stem cells (CSCs) which play a crucial role in resistance to chemotherapy and radiotherapy, providing a clear mechanism for relapse of after initial therapeutic treatment^7–9^. Most therapeutic strategies rely on using cytotoxic methods that induce apoptosis in rapidly dividing cells. Though this may injure other rapidly dividing non-cancerous cells, this type of therapy is effective in shrinking the size of many solid tumors, often reducing the bulk of the carcinoma beyond the limit of clinical detection. However, the cells that remain beyond this detection limit are usually CSCs, which are less susceptible to conventional treatment^10^.

There are a number of signaling mechanisms that regulate EMT induction. These include various signaling pathways, such as: TGF-β, Notch and WNT. These pathways help regulate the expression of EMT transcription factors such as: Snail, Slug, Twist, Zeb1/2. Additionally, RNA splicing, microRNA expression, DNA methylation and histone modifications also play important roles in EMT induction^1,11,12^. However, there has been little evidence regarding whether histone variants directly participate in EMT regulation.

The histone variant macroH2A1 is expressed in nearly all cell types and is involved in a number of processes including cell cycle, gene regulation, DNA damage repair and senescence^13–15^. The knockdown of macroH2A1 facilitates reprogramming of induced pluripotent stem cells derived from keratinocytes (KiPSCs) and its overexpression hampers the reprogramming process. Additionally, macroH2A1 knockdown in KiPSCs facilitates the restoration of epigenetic activating modification H3K4me2 at pluripotency genes during reprogramming. Also, macroH2A1 expression in self-renewing human embryonic stem cells (hESCs) was notably low. However upon spontaneous differentiation, the cells downregulate their Oct4 expression and upregulate macroH2A1^16^. These results, as well as findings of other studies, demonstrate macroH2A1 acting as a barrier to reprogramming, keeping mature cells in their differentiated state^16–19^.

Additionally, macroH2A1 plays a role in cancer biology, particularly in proliferation and metastasis^20–22^. The *H2AFY* gene (which encodes for macroH2A1) has two isoforms (macroH2A1.1 and macroH2A1.2), which are produced from alternative splicing of mutually exclusive exons. These isoforms have significant similarity to the canonical histone H2A; however, they are distinguished by their ~25 kDa carboxy-terminal globular macrodomains. MacroH2A1.1 is noted by its capacity to bind the NAD^+^ metabolite poly(ADP-ribose) (PAR). However, the macroH2A1.2-specific exon renders it incapable of binding PAR^23^. The ratio in which these isoforms are generated is important in the context of metastasis^24^, as reduced macroH2A1.1 has been indicative of more aggressive tumors, and has been used a prognostic factor for certain types of cancers^21,25^. However, increased macroH2A1.1 ratio paradoxically is correlated with poor prognosis of triple-negative breast cancer^26^. Thus, a clear understanding of the functional difference of macroH2A1 isoforms is needed for resolving these seemingly contrasting observations.

Histone variants are important for regulating a number of cellular processes, however little is known about how their properties affect EMT. Given the well-established role of macroH2A1 as a barrier to reprogramming, and its association with cancer progression, we postulated that macroH2A1 may play a role in EMT, a process involving reprogramming of cellular states and an initiator of metastasis. In this study, we demonstrate that ectopic expression of macroH2A1.1 can suppress EMT induction. This EMT suppression is mediated by macroH2A1.1’s ability to bind PAR.

## Results

### MacroH2A1 protein expression is reduced during EMT induction

**Figure 1:**
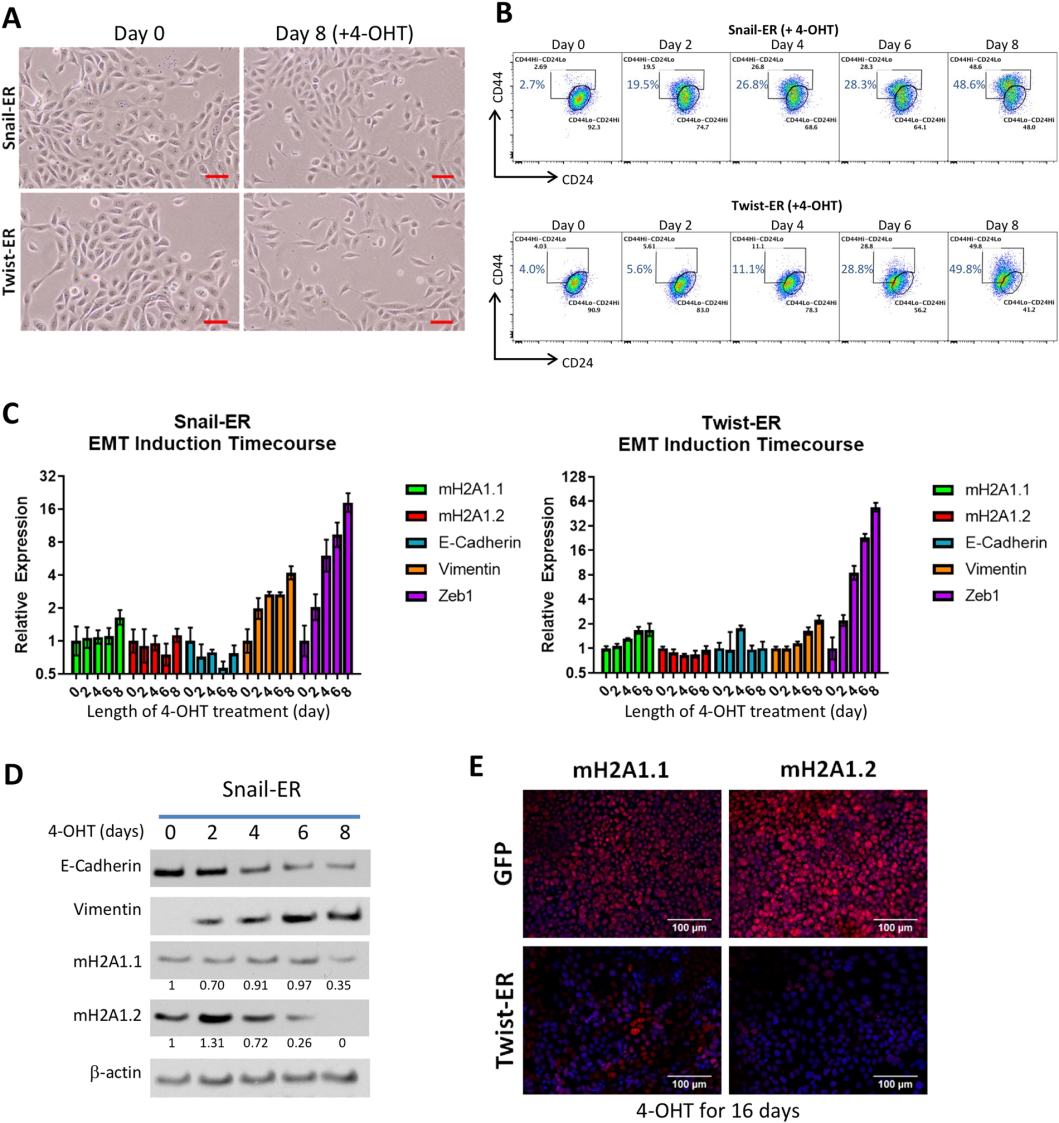
(**A**) Phase contrast image of HMLE cells expressing Snail-ER or Twist-ER at 0 and 8 days post 4-OHT treatment. Scale bar = 100 μm. (**B**) Flow cytometric profiles of CD44 and CD24 on Snail-ER or Twist-ER HMLE cells treated with 4-OHT for the indicated number of days. (**C**) Relative expression of the mRNAs encoding macroH2A1.1, macroH2A1.2, E-cadherin, vimentin, and Zeb1 in the cells described in (**B**) as determined by real-time RT-PCR. GAPDH was used as a housekeeping gene control to account for variability in template loading. (**D**) Western blot analysis of expression of E-cadherin, vimentin, macroH2A1.1 and macroH2A1.2, proteins in the HMLE-Snail-ER cells treated with 4-OHT for the indicated number of days. Histone H3 was used as a loading control. (**E**) Immunofluorescence images of HMLE-Twist-ER cells treated with 4-OHT for 16 days. Cells were stained using antibodies against macroH2A1.1, macroH2A1.2, E-cadherin, or vimentin. Scale bar = 100 μm.

EMT can be induced in immortalized mammary epithelial cells (HMLEs) with the overexpression of the transcription factors *SNAI1* (Snail) or *TWIST1* (Twist)^27^. We used two tamoxifen-inducible EMT cell lines expressing either Snail or Twist fused to a mutated estrogen receptor ligand-binding domain (Snail-ER or Twist-ER) to observe the kinetics of EMT induction in HMLEs^27^. After treatment of the HMLE-Snail-ER and HMLE-Twist-ER cells with 4-hydroxytamoxifen (4-OHT) for eight days, the HMLE cells exhibited phenotypes typical of EMT. The cell morphology began to change from its typical cuboidal/cobblestone-like appearance, to a more elongated, fibroblast-like shape (Fig. 1A). Cell surface markers CD44 and CD24 have been used to describe mesenchymal-like cells within the heterogeneous HMLEs^27^. Using flow cytometry, we observed an 18-fold increase in the CD44^Hi^/CD24^Lo^ population (indicative of cells having undergone EMT^27^) (Fig. 1B). During this time period, we examined the mRNA expression in the HMLE-Snail-ER and HMLE-Twist-ER cells via qRT-PCR. Our analysis focused on the levels of both of the macroH2A1 splice forms (macroH2A1.1 and macroH2A1.2), as well as for EMT-related genes (E-Cadherin, vimentin and Zeb1). At the end of eight days of treatment, mRNA levels of macroH2A1.1, macroH2A1.2, E-Cadherin remained relatively unchanged. Vimentin levels increased over four-fold and two-fold for HMLE-Snail-ER and HMLE-Twist-ER, respectively. The transcription factor Zeb1 (downstream of Snail and Twist in the EMT signaling cascade) showed a nearly 18 and 55-fold increase respectively for HMLE-Snail-ER and HMLE-Twist-ER, indicating that the EMT induction process was under way (Fig. 1C). This finding was corroborated by western blot of the HMLE-Snail-ER cells which demonstrated a significant decrease in E-Cadherin protein expression and nearly a two-fold increase in Vimentin protein expression.

Although the mRNA levels of macroH2A1.1 and macroH2A1.2 did not change significantly in the HMLE-Snail-ER cells during this eight-day process, western blot analysis clearly demonstrated a 65% reduction of macroH2A1.1 and a near complete loss of macroH2A1.2 protein expression by day 8 (Fig. 1D). In HMLE-Twist-ER cells that were treated with 4-OHT for 16 days, we observed similar results via immunofluorescence. We noted a nearly complete loss of E-Cadherin expression, demonstrating the progression of EMT. Importantly, we observed a substantial loss of expression of macroH2A1.1 and macroH2A1.2 (Fig. 1E). Overall, these data indicate that during EMT in HMLEs, macroH2A1 protein expression is dramatically reduced in a post-transcriptional manner. The regulation of macroH2A1 protein translation and stability remains incompletely understood. Previous work has shown that macroH2A1 levels are regulated by post-transcriptional mechanisms and that macroH2A1 is most stable when incorporated into nucleosomes and the unincorporated proteins are turned over rapidly^28,29^. Thus, it is possible that either the translation efficiency or chromatin occupancy of macroH2A1 is inhibited during EMT, hence causing the downregulation of the macroH2A1 proteins despite no change in mRNA levels. Future studies are needed to distinguish these two mechanisms.

### Ectopic macroH2A1.1 expression suppresses EMT induction

**Figure 2:**
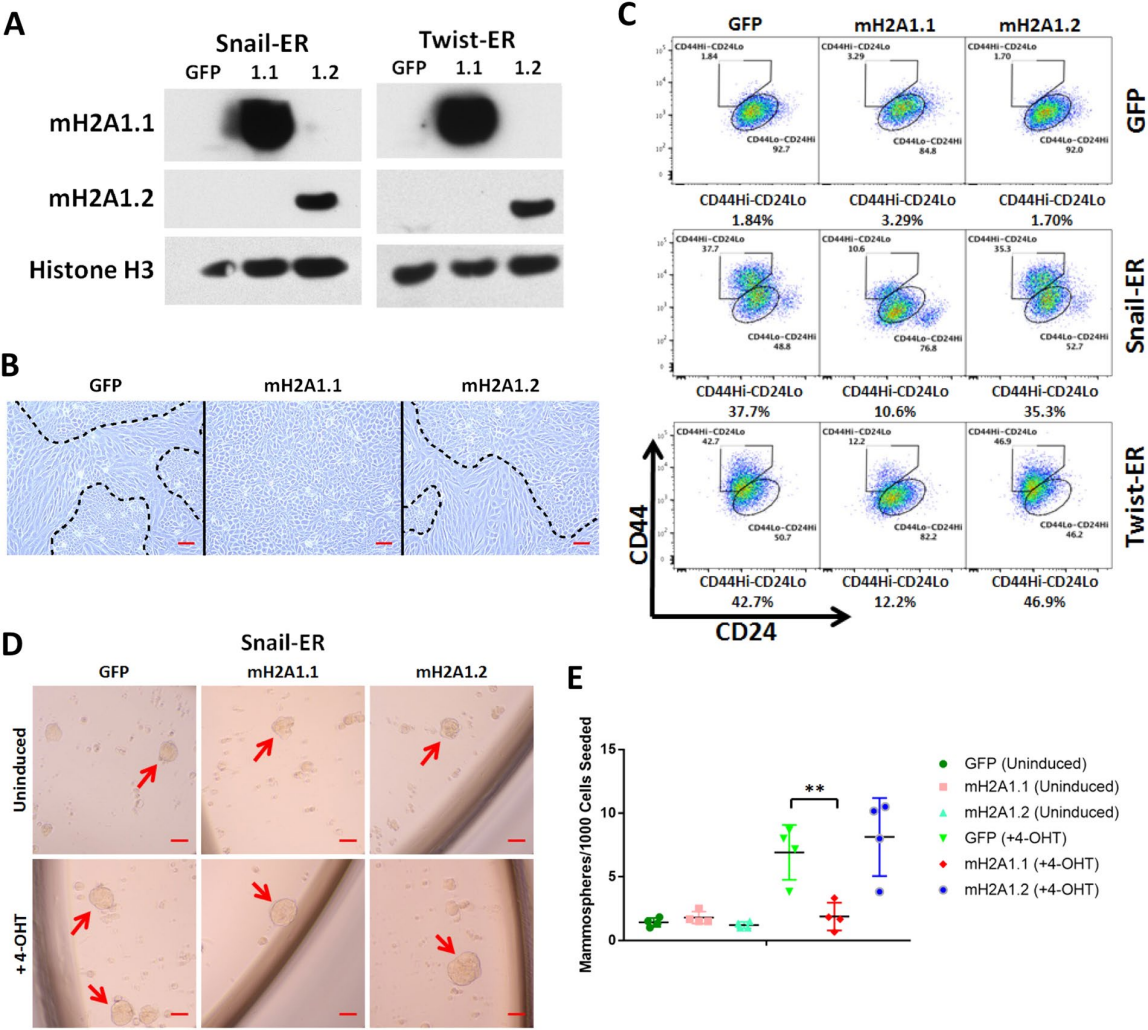
(**A**) Western blot analysis of expression of macroH2A1.1, macroH2A1.2 proteins in the HMLE-Snail-ER or HMLE-Twist-ER cells transduced with the indicated vectors. Histone H3 was used as a loading control. (**B**) Phase contrast of HMLE Twist-ER cells overexpressing macroH2A1.1 or macroH2A1.2 after 8-day 4-OHT treatment. Mesenchymal cell morphology was observed in the vector control and macroH2A1.2 cells, but not macroH2A1.1 cells. Scale bar = 100 μm. (**C**) Flow cytometric analysis of cell-surface markers, CD44 and CD24, in the indicated cells after 8-day 4-OHT treatment. (**D**) Phase-contrast images of mammospheres formed from HMLE-Snail-ER cells transduced with the indicated macroH2A1 vectors and treated ± 4-OHT for 8 days. Scale bar = 100 μm. (**E**) Quantification of mammospheres formed in (**D**). (n = 4 experiments, p = 0.005 (GFP vs. macroH2A1.1)).

The loss of macroH2A1 during EMT raises the question of whether this histone variant plays a functional role in the initiation of the process. To examine the role of each macroH2A1 isoform on EMT induction, we used a retroviral vector to ectopically express each macroH2A1 isoform in HMLE-Snail-ER and HMLE-Twist-ER cells (Fig. 2A). After eight days of 4-OHT treatment, we noticed that the cells underwent EMT in an expected fashion, with the exception of those cells ectopically expressing macroH2A1.1. The macroH2A1.1 overexpressing cells had a less pronounced mesenchymal morphology than those expressing the control vector or macroH2A1.2 (Fig. 2B). Measuring the cell profile via flow cytometry, we observed a 70 percent relative reduction of the CD44^Hi^/CD24^Lo^ population in the macroH2A1.1 cells (Fig. 2C).

In order to assess the stem-like property of the CD44^Hi^/CD24^Lo^ cells, we performed a 3D mammosphere culture assay, in which only cells with stem-like characteristics may grow into spheres^30^. When HMLE-Snail-ER cells with ectopic macroH2A1 expression were grown in mammosphere conditions to examine their self-renewing ability, we observed a near complete inhibition of the increase in mammosphere-forming ability by Snail activation in the cells overexpressing macroH2A1.1, compared to those with the vector control or overexpressing macroH2A1.2 (Fig. 2D and E). Interestingly, macroH2A1.1 overexpression did not affect the mammosphere-forming ability of uninduced HMLE-Snail-ER cells, suggesting that its inhibitory effect on mammosphere-forming cell induction is not due to general cytostatic effects. This finding corroborates the previous data showing a significant decrease in the CD44^Hi^/CD24^Lo^ population and the maintenance of epithelial morphology in macroH2A1.1-expressing cells. Together, they suggest that ectopic expression of macroH2A1.1, but not macroH2A1.2 suppresses the induction of EMT and the associated stem cell-like self-renewal property.

### Knockout of macroH2A1.1 alone is not sufficient to induce EMT

**Figure 3:**
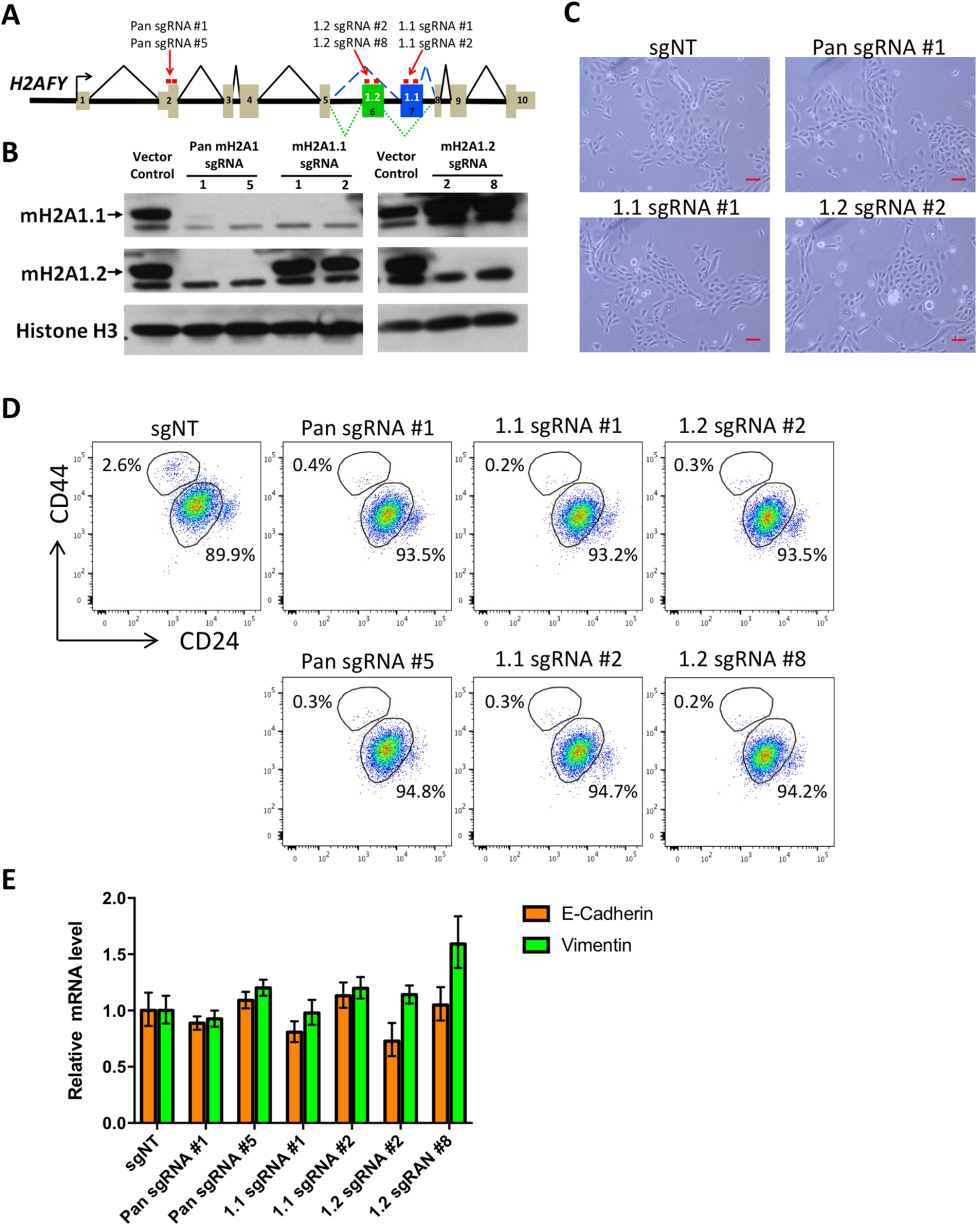
(**A**) Schematic diagram showing the targeting sites of macroH2A1 sgRNAs. (**B**) Western blot measuring macroH2A1 proteins in HMLE Snail-ER cells transduced with the indicated lentiCRISPRv2 vectors. Cells were analyzed 16 days after transduction. (**C**) Phase-contrast images showing the representative morphology of HMLE Snail-ER cells transduced with the indicated lentiCRISPRv2 vectors. Cells were imaged 16 days after transduction. Scale bar = 100 μm. (**D**) Flow cytometric analysis measuring CD44 and CD24 profiles in HMLE Snail-ER cells transduced with the indicated lentiCRISPRv2 vectors. Cells were analyzed 16 days after transduction. (**E**) E-cadherin and Vimentin mRNA levels in HMLE Snail-ER cells transduced by the indicated lentiCRISPRv2 vectors. Cells were analyzed by qRT-PCR 16 days after transduction. *GAPDH* was used as a housekeeping gene control.

Given the profound effect of macroH2A1.1 overexpression in EMT inhibition, we asked whether downregulating endogenous macroH2A1.1 is sufficient to induce EMT by itself. Specific knockdown of specific macroH2A1 isoforms has been difficult due to lack of effective shRNA targeting isoform-specific exons. Thus, we resorted to CRISPR-mediated knockout by using a constitutive lentiviral CRISPR vector (lentiCRISPRv2)^31^. We were able to identify effective sgRNAs targeting *H2AFY* (encoding macroH2A1) exons that are either specific to individual isoform or shared by both isoforms (Fig. 3A). Transduction of HMLE cells with these lentiviral CRISPR vectors yielded specific ablation of intended protein variants (Fig. 3B). This allowed us to investigate the effect of the loss of each individual variant as well as their combined loss on EMT induction. Interestingly, neither knockout of individual macroH2A1 variants or the entire gene were sufficient to induce EMT in HMLE cells, as measured by cell morphology (Fig. 3C), the CD44/CD24 profile (Fig. 3D), and E-cadherin and vimentin expression (Fig. 3E). This leads us to believe that macroH2A1.1 loss is not sufficient to initiate EMT, rather it is likely to act as a barrier to EMT reprogramming induced by other EMT inducers. Of note, the effective isoform-specific CRISPR developed here will be of significant value for studying isoform-specific functions of macroH2A1 in other biological processes.

### Ectopic macroH2A1 expression is not sufficient to cause MET in fully mesenchymal cells

**Figure 4:**
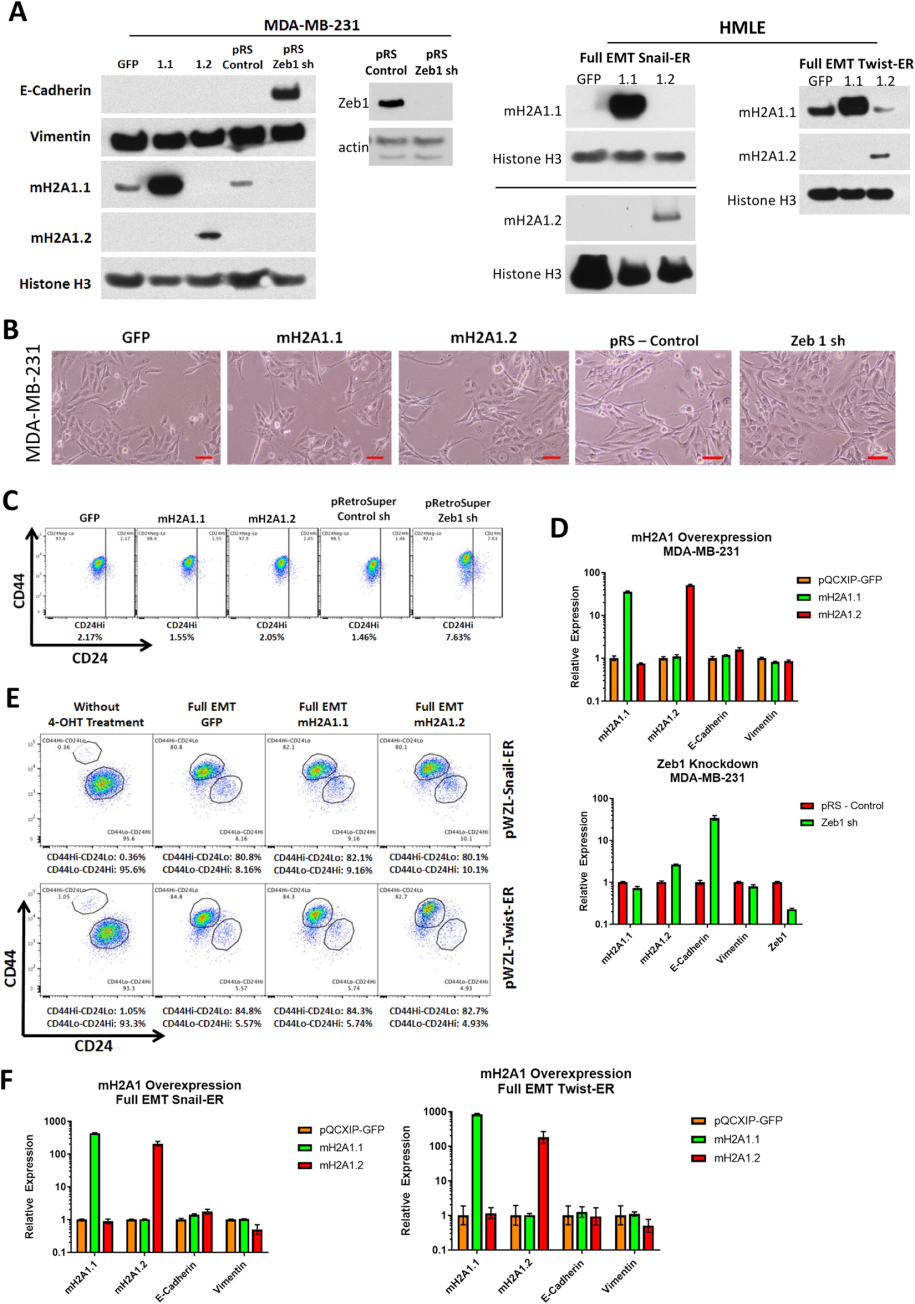
(**A**) Western blot analysis of expression of MDA-MB-231, HMLE-Snail-ER and HMLE-Twist-ER cells transduced by the indicated vectors. Histone H3 was used as a loading control. (**B**) Phase contrast of MDA-MB-231 cells overexpressing macroH2A1.1, macroH2A1.2 or Zeb1 shRNA. Scale bar = 100 μm. (**C**) Flow cytometric analysis of cell-surface markers, CD44 and CD24, in the cells described in (**B**). (**D**) Relative expression of the mRNAs encoding macroH2A1.1, macroH2A1.2, E-cadherin, vimentin, and Zeb1 in the cells described in (**B**) as determined by real-time RT-PCR. Normalized to GAPDH mRNA to account for variability in template loading. (**E**) Flow cytometric analysis of cell-surface markers, CD44 and CD24, in fully mesenchymal HMLE-Snail-ER or HMLE-Twist-ER cells overexpressing macroH2A1.1, macroH2A1.2 or the control vector. (**F**) Relative expression of the mRNAs encoding macroH2A1.1, macroH2A1.2, E-cadherin, vimentin, and Zeb1 in the cells described in (**E**) as determined by real-time RT-PCR. Normalized to GAPDH mRNA to account for variability in template loading.

Our previous experiments analyzed the effect of macroH2A1 during the initiation and progression of EMT. While the ectopic expression of macroH2A1.1 does suppress the transition of HMLEs to the mesenchymal state, we sought to illuminate whether ectopic macroH2A1 can initiate a reversal of the process: induction of MET. To examine this, we decided to use MDA-MB-231 cells, a metastatic breast cancer cell line that expresses high levels of Snail and Twist^32,33^. MDA-MB-231 cells are mesenchymal, having completely undergone the transition from their previous epithelial state. In order to determine whether high levels of macroH2A1 could induce MET, we ectopically expressed macroH2A1.1 and macroH2A1.2 in these cells and examined if there were any changes in their morphology, antigenic surface markers, or E-Cadherin and vimentin expression (Fig. 4A). As a positive control, we knocked down the transcription factor Zeb1 via shRNA, which has been shown to initiate MET^34^. Similar to what has been previously reported, Zeb1 knockdown in MDA-MB-231 did cause a significant upregulation of E-Cadherin expression (Fig. 4A and D), however ectopic expression of macroH2A1.1 and macroH2A1.2 did not affect MDA-MB-231 cell morphology, antigenic surface markers or E-Cadherin or vimentin expression levels (Fig. 4B–D).

To rule out the possibility that the requirement of macroH2A1 in EMT in MDA-MB-231 cells has already been bypassed by certain genetic mutations in these cancer cells, we also generated stable HMLE mesenchymal cells (whose EMT induction is regulated by macroH2A1), by treating HMLE-Snail-ER and HMLE-Twist-ER cells with 4-OHT for 16 days and FACS sorted the resulting CD44^Hi^/CD24^Lo^ cells. These cells had already undergone transition from an epithelial to a mesenchymal state and can maintain a stable mesenchymal phenotype in culture (Fig. 4E). We ectopically expressed macroH2A1 isoforms in these cells then analyzed them for changes in their CD44^Hi^/CD24^Lo^ profile after ectopic expression, however there were no changes observed compared to the vector control (Fig. 4A and E). We also examined these cells via qRT-PCR and we did not observe any changes in EMT markers relative to the vector control (Fig. 4F). This leads us to conclude that while macroH2A1 plays a role during the initial phases of EMT, and its ectopic expression can hamper progression towards a mesenchymal state, ectopic macroH2A1 alone is not sufficient to cause mesenchymal cells to undergo MET. Thus, while macroH2A1.1 can block the ability of HMLE cells to undergo the transition to the mesenchymal state, this data suggest that reduced expression of macroH2A1.1 is not required to maintain cells in a mesenchymal state. However, whether macroH2A1 can cooperate with other MET-inducing signals (such as Protein Kinase A activation^35^) requires future investigation.

### The PAR Binding Activity of MacroH2A1.1 Is Required for EMT Suppression

**Figure 5:**
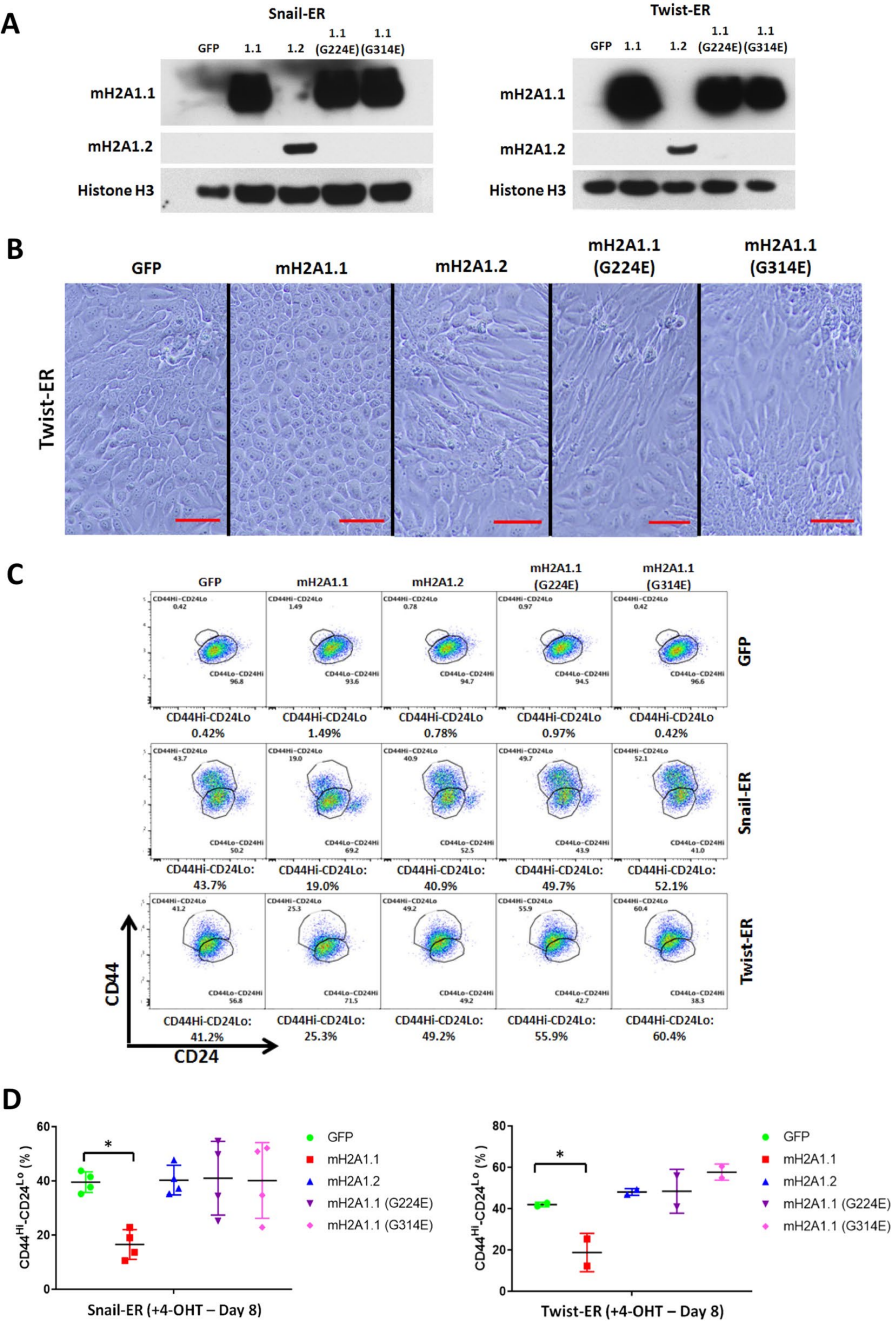
(**A**) Western blot analysis of expression of macroH2A1.1, macroH2A1.2 proteins in the HMLE-Snail-ER or HMLE-Twist-ER cells transduced by the indicated retroviral vectors. Histone H3 was used as a loading control. (**A**,**B**) Phase contrast of HMLE-Twist-ER cells overexpressing macroH2A1.1, macroH2A1.2, mutant macroH2A1.1 (G224E and G314E) after 8-day 4-OHT treatment. Mesenchymal morphology observed in the vector control, macroH2A1.2 cells and mutant macroH2A1.1 cells, but not macroH2A1.1 cells. Scale bar = 100 μm. (**B**,**C**) Flow cytometric analysis of cell-surface markers, CD44 and CD24, in the indicated cells treated with 4-OHT for 8 days. (**C**,**D**) Quantification of HMLE-Snail-ER and HMLE-Twist-ER cells as described in (**C**). (n = 4 experiments for Snail-ER (p = 0.0129), 2 experiments for Twist-ER (p = 0.0478) – GFP vs. macroH2A1.1)).

We further investigated the mechanism involved in the isoform-specific function of macroH2A1 in EMT. EMT suppression was only observed in the macroH2A1.1 overexpressing cells. This gave us an indication about a possible mechanism, given that the major distinction of macroH2A1 isoforms is that macroH2A1.1 can bind PAR and macroH2A1.2 is incapable of doing so. In order to test whether this PAR binding ability was responsible for the observed EMT suppression, we overexpressed two variants of macroH2A1.1 with point mutations (G224E and G314E) that abolish its ability to bind PAR^23,28,29,36^ (Fig. 5A).

After treating the HMLE-Snail-ER and HMLE-Twist-ER cells with 4-OHT for eight days, the same phenotype of EMT suppression was again observed in the wild-type macroH2A1.1 overexpressing cells. In the HMLE-Twist-ER cells, it appeared that macroH2A1.1’s ectopic expression allowed for the HMLEs to maintain an epithelial phenotype, whereas those overexpressing macroH2A1.2 or the mutant macroH2A1.1 displayed more elongated cells, indicative of more mesenchymal-like features (Fig. 5B). Similar to Fig. 2C, ectopic macroH2A1.1 suppressed the induction of CD44^Hi^/CD24^Lo^ cells. However, in the cells expressing macroH2A1.1 mutants that are unable to bind PAR, the suppressing effect was completely abolished (Fig. 5C and D) indicating that PAR binding is essential for macroH2A1.1’s ability to suppress EMT progression.

## Discussion

EMT is a dynamic cellular program that is involved in a number of biological processes. It can be regulated by multiple signaling pathways; however, the effects of histone variants on its regulation have yet to be fully explored. The results presented demonstrate that certain histone variants play an important role controlling the progression of EMT. We found that the macroH2A1.1 isoform, but not macroH2A1.2 isoform, acts as an inhibitor of EMT induction, and reduces stem cell activity, demonstrating a novel isoform-specific function of macroH2A1 in EMT. Another recent study corroborates the importance in macroH2A1 in EMT. By knocking down macroH2A1 expression via shRNAs in LD611 bladder cancer cells, these cells exhibited enhanced migration, invasion and self-renewal capacity, although this study did not investigate the isoform-specific function^20^.

We found the isoform-specific function in EMT can be attributed to the unique NAD^+^ metabolite PAR binding ability of macroH2A1.1. This has interesting implications in the context of EMT, as macroH2A1.1 has been shown to increase expression during the differentiation of tissues^21^ and given its role as prognostic factor for cancer^21,25,37^. The suppression of EMT by macroH2A1.1 seems to align with one of its functions, which is the reduction of PARP-1 protein expression^29^. PARP-1 has been shown to increase levels of Snail which represses E-Cadherin, and allows for EMT progression^38,39^. Ectopic expression of macroH2A1.1 in HMLEs undergoing EMT (by overexpressing Snail or Twist) could greatly reduce their PARP-1 activity, thereby limiting its role in upregulating Snail transcription, and reducing post-translational stabilization of its protein, thus suppressing EMT. Consequently, mutating Gly224 and Gly314 to Glu renders macroH2A1.1 unable to bind PAR, which could allow for Snail to continue to be stabilized, allowing for EMT to progress.

It has already been shown that PARP-1 inhibition can potentiate the effects of chemotherapy in triple-negative breast cancer^40^, as well as improve progression-free survival for patients with ovarian cancer sensitive to platinum-based agents^41,42^. Given the role of EMT in metastasis, and macroH2A1.1’s role in inhibiting PARP-1 activity, our findings could provide insight into understanding EMT progression, and potentially provide a new mechanism as to how macroH2A1 expression affects cancer progression.

The data presented provide additional support for the role of histone variants in regulating EMT^43^. Understanding the complexities of this process is important considering the wide range of biological scenarios in which EMT is involved. We also implicate an interaction between histone variants and transcription factors in the regulation of EMT. Our findings add to the growing list of factors that provide control over this important cellular process.

## Methods

### Cell Culture

Immortalized human mammary epithelial cells (HMLE) were maintained in media containing MEGM and DMEM/F-12 (1:1) supplemented with EGF, insulin and hydrocortisone. For 4-hydroxy tamoxifen (4-OHT) treatment, the HMLE-Snail-ER or HMLE-Twist-ER cells were treated with a final concentration of 100 nM for the indicated number of days. During treatment, medium was refreshed every two days.

### Retroviral Vectors

HMLE-Snail-ER and HMLE-Twist-ER were generated by retroviral gene transduction using the pWZL retroviral vector as previously published^27^, followed by selection with blasticidin (5 μg/mL). Ectopic macroH2A1 expression in HMLE-Snail-ER, HMLE-Twist-ER and MDA-MB-231 cells were created via gene transduction using the pQCXIP retroviral vector expressing Green Fluorescent Protein (GFP) as a control, macroH2A1.1, macroH2A1.2, or macroH2A1.1 point mutants G224E or G314E followed by puromycin (2 μg/mL) drug selection^23,28,29,36^. The pRetroSuper Zeb1 shRNA and control vectors were kind gifts from Dr. Thomas Brabletz^34^. They were used to transduce MDA-MB-231 cells followed by puromycin (2 μg/mL) drug selection.

### CRISPR-mediated macroH2A1 knockout

The sgRNA sequences were designed using the CRISPR Design Tool from the Zhang Laboratory (http://crispr.mit.edu) and cloned into the lentiCRISPRv2 vector (Addgene # 52961, a gift from Feng Zhang). HMLE cells were transduced with these vectors and selected by puromycin. The sgRNA targeting sequences are as the following: pan sgRNA #1 – GCCACCGCCATGTCGAGCCG, pan sgRNA #2 – CGTGTACATGGCCGCCGTCC, 1.1 sgRNA #1 – GACAGCATCACTGTCGATCG, 1.1 sgRNA #2 – AACACTGACTTCTACATCGG, 1.2 sgRNA #2 – TAATTTAGCCGGCTTTGAGG, 1.2 sgRNA #8 – TTTAAGGTCAATGTCAGCAT, and sgNT – GCGAGGTATTCGGCTCCGCG.

### Antibodies and Western Blotting

Cells were lysed with the lysis buffer containing 50 mM Tris, pH 8.0, 150 mM NaCl, 0.5% sodium deoxycholate, and 1.0% NP-40 on ice. 10 micrograms of total protein from each sample was resolved on a 4–12% Bis-Tris Gel with MOPs Running Buffer and transferred to PVDF membranes. The blots were then probed with various antibodies, including anti-E-cadherin (BD Biosciences – BDB610182), anti-Vimentin (Cell Signaling Technology – 5741S), anti- macroH2A1.1 (Cell Signaling Technology – 12455S), anti- macroH2A1.2 (Cell Signaling Technology – 4827S) or anti-Histone H3 (Cell Signaling Technology – 14269S). Original scans of the western blot results are provided in the Supplementary Information (Figure S1).

### Immunofluorescence

10 × 10^3^ cells were seeded on an 8-well Lab-TekII Chamber Slide. After 24 hours, the cells were washed with phosphate-buffered saline (PBS) twice, fixed in 10% formalin and permeabilized 0.1% Triton X100 in PBS buffer at room temperature for ten minutes. The cells were then washed three times with PBS and incubated with the blocking solution (10% goat serum in PBS). The cells were then incubated with the primary antibodies overnight, washed three times with PBS plus 0.1% Tween-20 for 15 minutes, and finally incubated with secondary antibodies (Invitrogen) for 30 minutes. The slides were washed extensively with PBS and mounted with DAPI Fluoromount-G (SouthernBiotech – 0100-20). All matched samples were photographed (control and test) using immunofluorescence microscope and identical exposure times.

### Mammosphere Culture and Differentiation

Mammosphere culture was performed as described in Dontu *et al*.^30^.

### Reverse Transcriptase PCR Analysis

SYBR-Green real-time RT-PCR and the corresponding data analysis were conducted with the Comparative Ct Method (ΔΔCt). For all RT-PCR analysis GAPDH mRNA was used to normalize RNA inputs. The primers used are as follows:

GAPDH (Forward: CCCAGAAGACTGTGGATGG)

GAPDH (Reverse: CTGGACTGGACGGCAGATCT)

MacroH2A1.1/MacroH2A1.2 (Common Forward: GGCTTCACTGTCCTCTCCAC)

MacroH2A1.1 (Reverse: GGTGAACGACAGCATCACTG)

MacroH2A1.2 (Reverse: GGATTGATTATGGCCCTCCAC)

E-Cadherin (Forward: TGCCCAGAAAATGAAAAAGG)

E-Cadherin (Reverse: CTTGCGTAACGGTGTATGTG)

Vimentin (Forward: GAGAACTTTGCCGTTGAAGC)

Vimentin (Reverse: CTAACGGTGGATGTCCTTCG)

Zeb1: (Forward: ACGCAGTCTGGGTGTAATC)

Zeb1: (Reverse: GGGCATTCATATGGCTTCTCTC)

### Flow Cytometry

The anti-CD44 (clone G44-26) and anti-CD24 (clone ML5) antibodies used for FACS analysis were obtained from BD Bioscience.

### Statistical Analysis

All means were compared to one another and significance was determined by a one-way ANOVA using the Tukey multiple comparison test in GraphPad PRISM version 7.01.

## Electronic supplementary material

Supplementary Information
