# Supplementary material for: Histone Variant MacroH2A1 Plays an Isoform-Specific Role in Suppressing Epithelial-Mesenchymal Transition
Source: Sci Rep. 2018 Jan 16;8:841. doi: 10.1038/s41598-018-19364-4 (PMC5770377; doi:10.1038/s41598-018-19364-4)
Supplement: Supplementary file 1 — Supplementary Information [file 41598_2018_19364_MOESM1_ESM.pdf]

Histone Variant MacroH2A1 Plays an Isoform-Specific Role in Suppressing Epithelial-Mesenchymal Transition

Dayle Q. Hodge, Jihong Cui, Matthew J. Gamble,

Wenjun Guo

Supplementary Information

Supplementary Information

Figure 1D

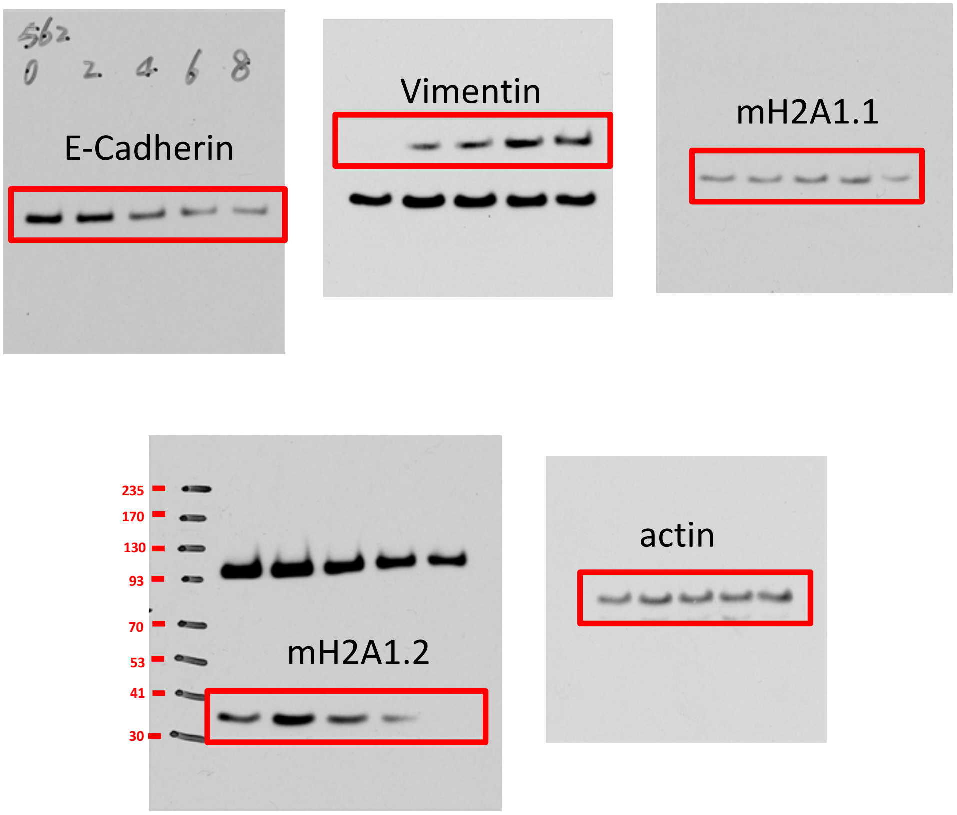

Figure 2A – Left panel (Snail-ER)

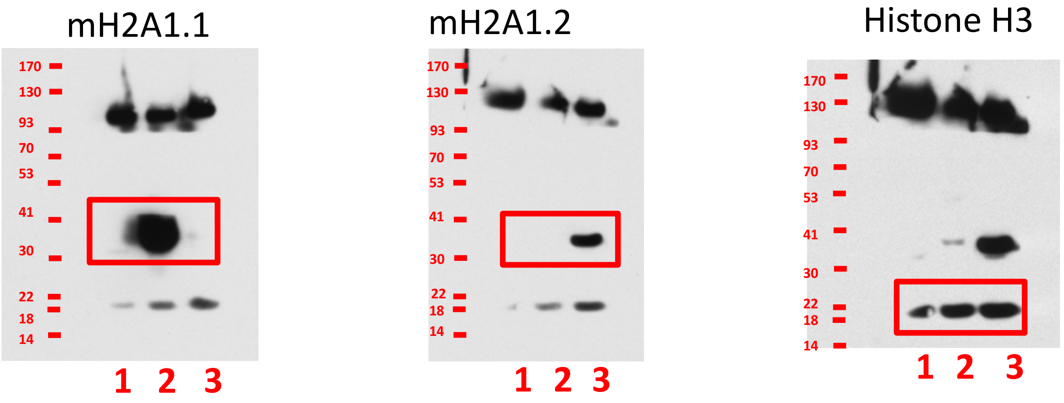

Figure S1 Western blot scanned films. Boxes highlight cropped films used in the figures.

Figure 2A – Right panel (Twist-ER)

mH2A1.1

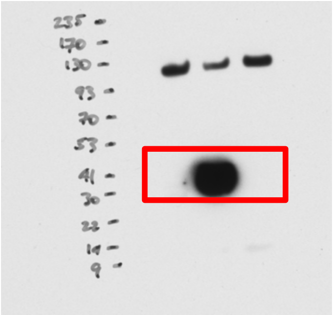

mH2A1.2

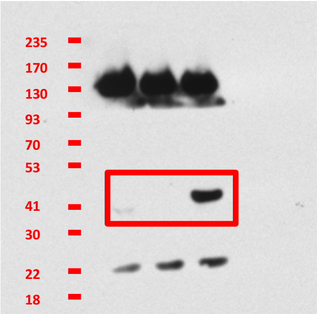

Histone H3

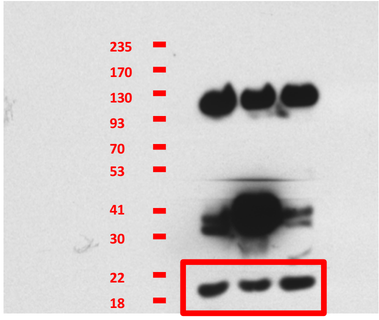

Figure 3B – Left Panel

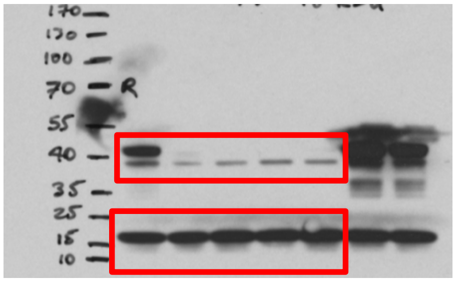

mH2A1.1

Histone H3

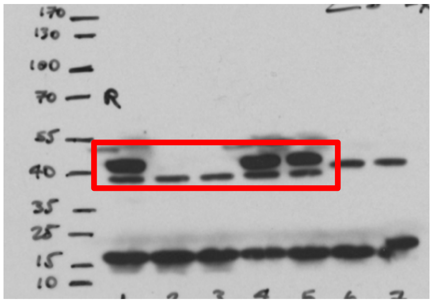

mH2A1.2

Figure 3B – Right Panel

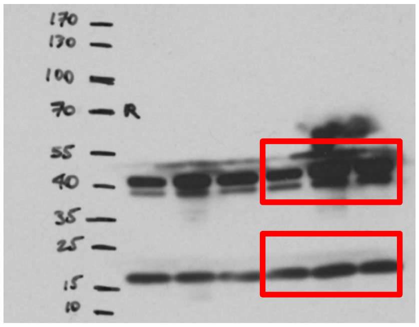

mH2A1.1

Histone H3

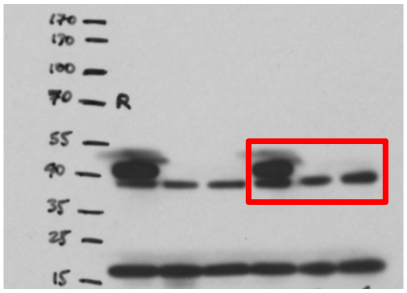

mH2A1.2

Figure S1 Continued.

Figure 4A – MDA-MB-231

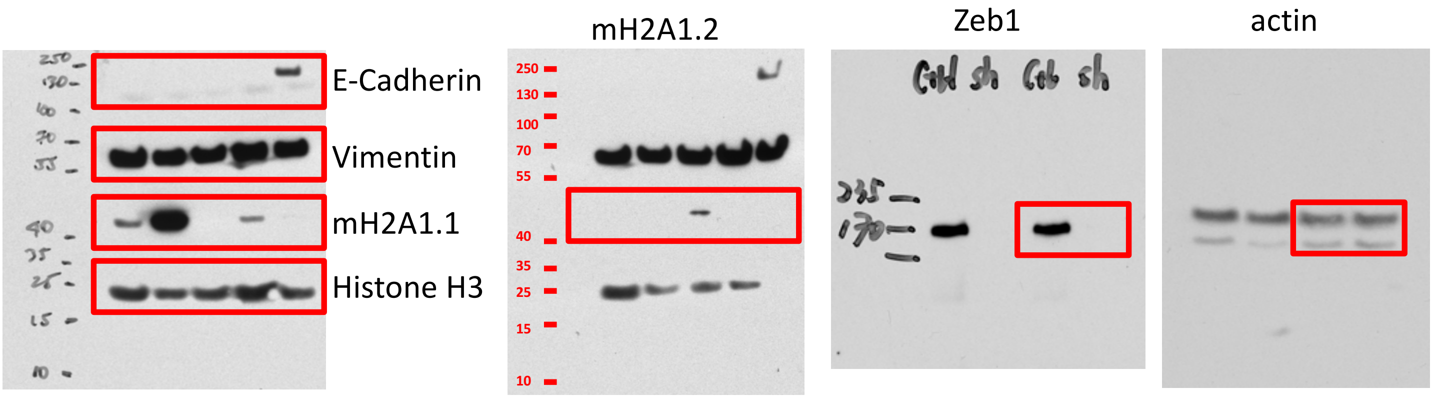

Figure 4A – Full EMT SnailER

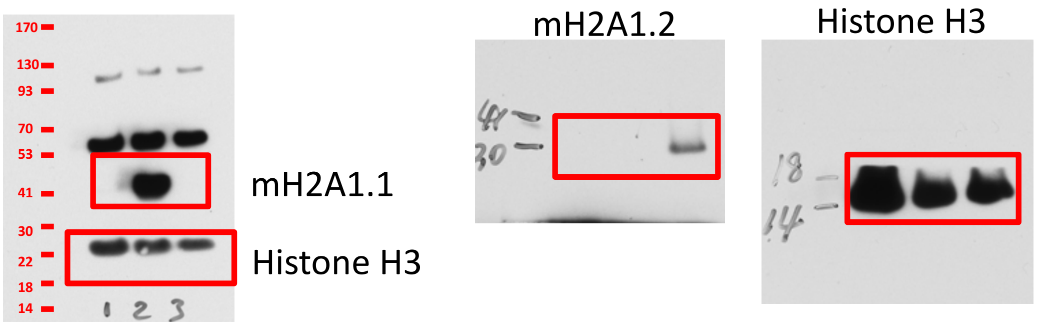

Figure 4A – Full EMT Twist-ER

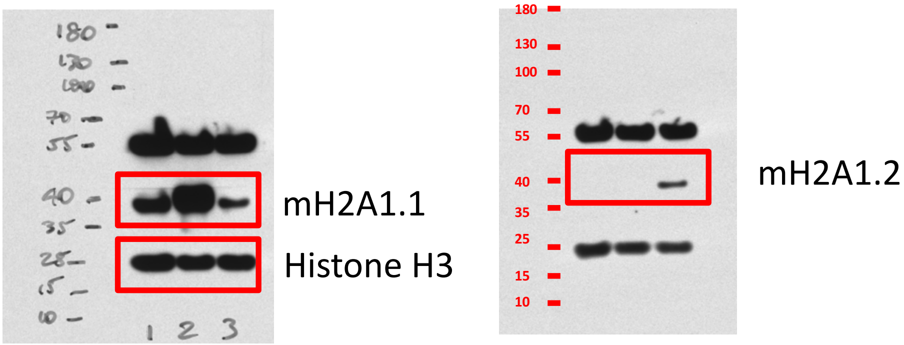

Figure S1 Continued.

Figure 5A – Left panel

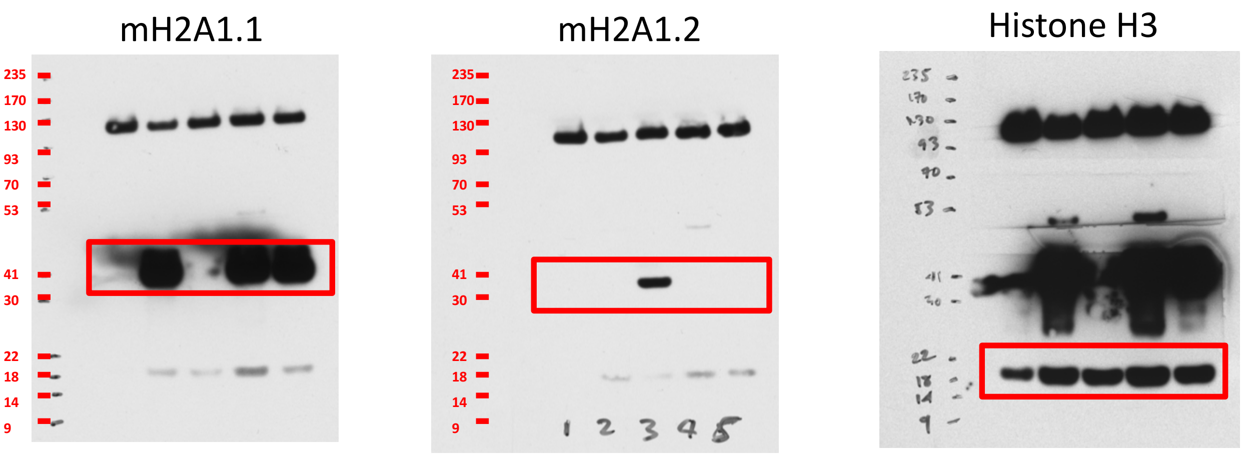

Figure 5A – Right panel

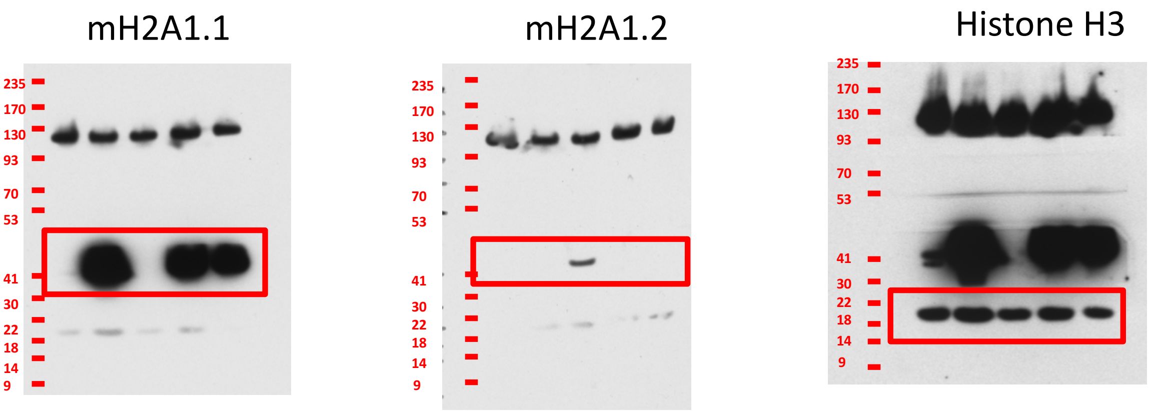

Figure S1 Continued.
